# Glissonean pedicle approach in liver surgery

**DOI:** 10.1002/ags3.12062

**Published:** 2018-02-13

**Authors:** Masakazu Yamamoto, Shun‐ichi Ariizumi

**Affiliations:** ^1^ Department of Surgery Institute of Gastroenterology Tokyo Women's Medical University Tokyo Japan

**Keywords:** anatomical hepatectomy, Laennec's capsule, surgical anatomy of the liver, the hilar plate, Walaeus sheath

## Abstract

Glisson's capsule was discovered by Johannis Walaeus in 1640 and described by Francis Glisson in 1654. The capsule wraps the hepatic artery, the portal vein and the bile duct in the liver and forms bundles at the hepatic hilus and in the liver as the Glissonean pedicle tree. Glisson's capsule does not connect to the proper membrane of the liver, which was discovered by Laennec; therefore, the Glissonean pedicles can be detached from the liver parenchyma without liver dissection. Couinaud described three main approaches to control the inflow system at the hepatic hilus in liver surgery; the intrafascial approach, the extrafascial and transfissural approach, and the extrafascial approach. The intrafascial approach is the so‐called control method. The extrafascial and transfissural approach, and the extrafascial approach are recognized as the Glissonean pedicle approach. When the Glissonean pedicles are ligated before liver transection, various types of anatomical hepatectomy can be carried out. The Glissonean pedicle approach is, therefore, considered to be one of the most important procedures in liver surgery. We herein describe the historical aspects and procedures of the Glissonean pedicle approach in liver surgery.

## 1 INTRODUCTION

Couinaud was convinced that Glisson's capsule was the most important component of the liver in his book entitled *Surgical Anatomy of the Liver Revisited*.^1^ The portal vein, the hepatic artery and the bile duct are wrapped in a connective tissue (Glisson's capsule) which accompanies them up to the liver parenchyma. This connective tissue forms a thick plate at the hepatic hilus. The plate is referred to as the hilar plate which connects the cystic plate, the umbilical plate and the Arantian plate. Glisson's capsule together with the artery, the portal vein and the bile duct forms a bundle of vessels which is referred to as the Glissonean (Glissonian, Glisson's) pedicle. The Glissonean pedicles can be separated easily when the hilar plate is detached from the liver parenchyma. Another important aspect of the liver surgical anatomy is that any variations of vascular and biliary elements usually occur under the plate system, so that dissection of the Glissonean pedicles above the plate system is considered to be low risk for injury to the elements of the remnant liver. The plate system of the liver has been recognized to be important from a surgical point of view.^1^

**Figure 1:**
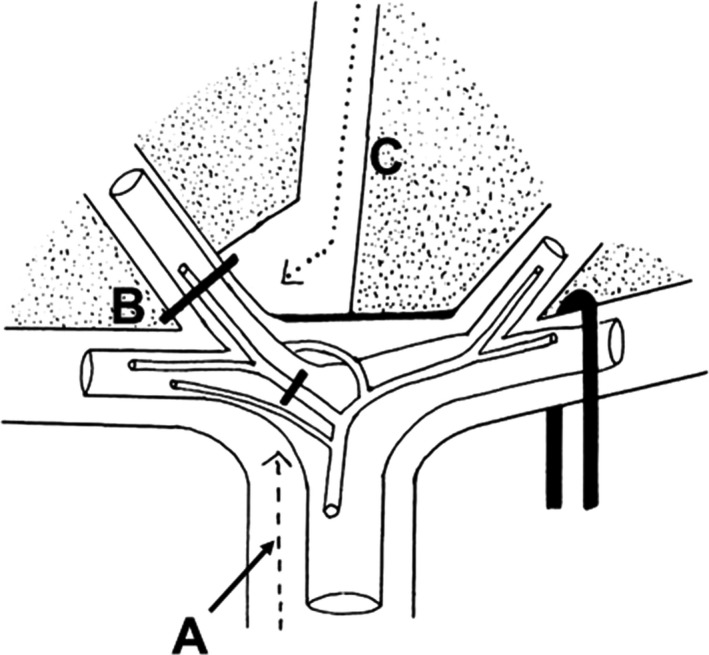
The three methods of access to the portal pedicle. A, Intrafascial approach. B, Extrafascial approach. C, Extrafascial and transfissural approach

Couinaud described three main approaches to the inflow system at the hepatic hilus; the intrafascial, the extrafascial, and the extrafascial and transfissural approach (Fig. 1).^1^ The extrafascial approach, and the extrafascial and transfissural approach are considered to be the Glissonean pedicle approach; therefore, we describe the details of the extrafascial approach, and the extrafascial and transfissural approach.

The Glissonean pedicle approach is available in laparoscopic hepatectomy and provides new knowledge of the surgical anatomy, especially for central hepatectomy and small anatomical liver resection less than sectionectomy and segmentectomy in the cirrhotic liver.^2^

## 2 THE INTRAFASCIAL APPROACH

The conventional dissection for separating the elements in the hepatoduodenal ligament is referred to as the intrafascial approach. The intrafascial approach is the so‐called “control method” which was introduced for right hepatectomy by Honjo and Lortat‐Jacob in the early 1950s.^3^, ^4^ Bismuth referred to this technique as the preliminary vascular control method (Lortat‐Jacob's technique).^5^ Bismuth, Fortner and Blumgart did not refer to Honjo's work in their articles.^5^, ^6^ However, Foster and Berman, and Helling introduced Honjo as predating Lortat‐Jacob.^7^, ^8^ Pack and Quattlebaum carried out right hepatectomy after primary hilar ligation in 1952 and 1953.^9^, ^10^ After Honjo and Lortat‐Jacob, the intrafascial approach was recognized as a standard procedure of anatomical hemi‐hepatectomy. Honjo, Lortat‐Jacob, Pack and Quattlebaum made tremendous contributions to liver surgery in the modern era. However, Bismuth and Couinaud pointed out that the intrafascial approach posed a risk of injuring the vessels and the bile ducts of the remaining liver as a result of wrong identification of several anatomical variations.^1^, ^5^ This is an important point in hepatobiliary surgery.

## 3 THE EXTRAFASCIAL AND TRANSFISSURAL APPROACH

If the main portal fissure or the left suprahepatic fissure is opened after liver parenchyma dissection, the surgeon can confirm the Glissonean pedicles which arise from the hilar plate or the umbilical plate. This procedure is referred to as the extrafascial and transfissural approach. This approach is considered to have been introduced by Ton That Tung in Vietnam and Tien‐Yu Lin in Taiwan around 1960.^11^, ^12^ Bismuth referred to this technique as the primary parenchymatous transection method (Ton That Tung's technique).^5^

**Figure 2:**
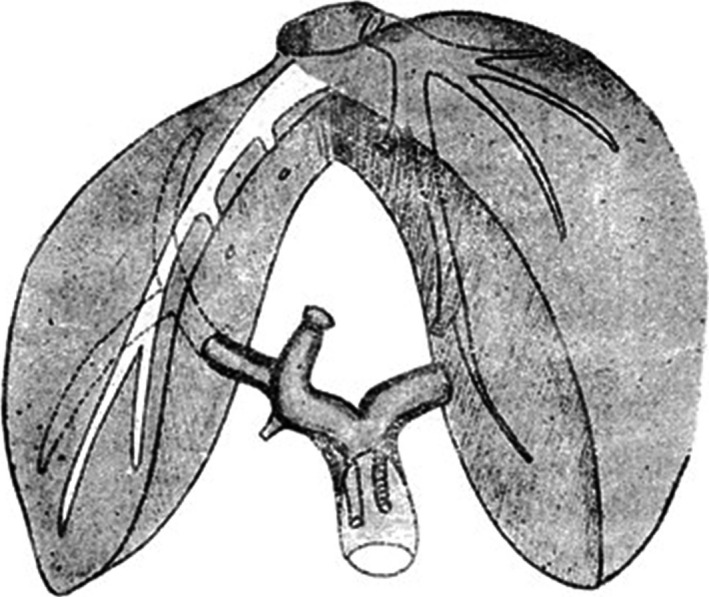
Anterior sectionectomy with the extrafascial and transfissural approach by Ton That Tung (Reprinted from reference^13^)

Ton That Tung was a very famous hepatic surgeon and surgical anatomist. He published three textbooks of liver surgery in Vietnamese and French. He described a large number of anatomical hepatectomies using the extrafascial and transfissural approach in 1971 (Fig. 2).^13^ Helling stated that Ton That Tung remains a pioneer in liver anatomy and liver surgery. However, his contributions were overshadowed by the national struggles of the Vietnamese to establish national independence. His understanding of liver anatomy based on meticulous dissection of autopsy specimens antedated and rivaled later works by Couinaud, Healey, Schroy and others.^8^

Tien‐Yu Lin in Taiwan also reported the extrafascial and transfissural approach with the finger fracture method and a special hepatic clamp.^14^ Lin described how the vascular and ductal components could be divided during liver transection. Lin did not use the term “Glissonean pedicle” in his article; however, Lin's procedure should be recognized as the extrafascial and transfissural approach to the intrahepatic Glissonean pedicles.^14^

In Japan, Okamoto also reported the extrafascial and transfissural approach in 1986. Okamoto transected the liver parenchyma of segment IV along the hilar plate and approached the anterior sectional pedicle of the right liver. Okamoto named this procedure the “unroofing method”. Okamoto also described how anatomical segmentectomy 5 and 8 could be carried out with the unroofing method.^15^

## 4 THE EXTRAFASCIAL APPROACH

The extrafascial approach was introduced by Takasaki and Couinaud around 1985. The extrafascial approach constitutes an approach to the pedicles at the hepatic hilus without liver dissection. When the hilar plate is pulled down after detaching the liver parenchyma, the right and left Glissonean pedicles can easily be approached.^1^, ^2^

**Figure 3:**
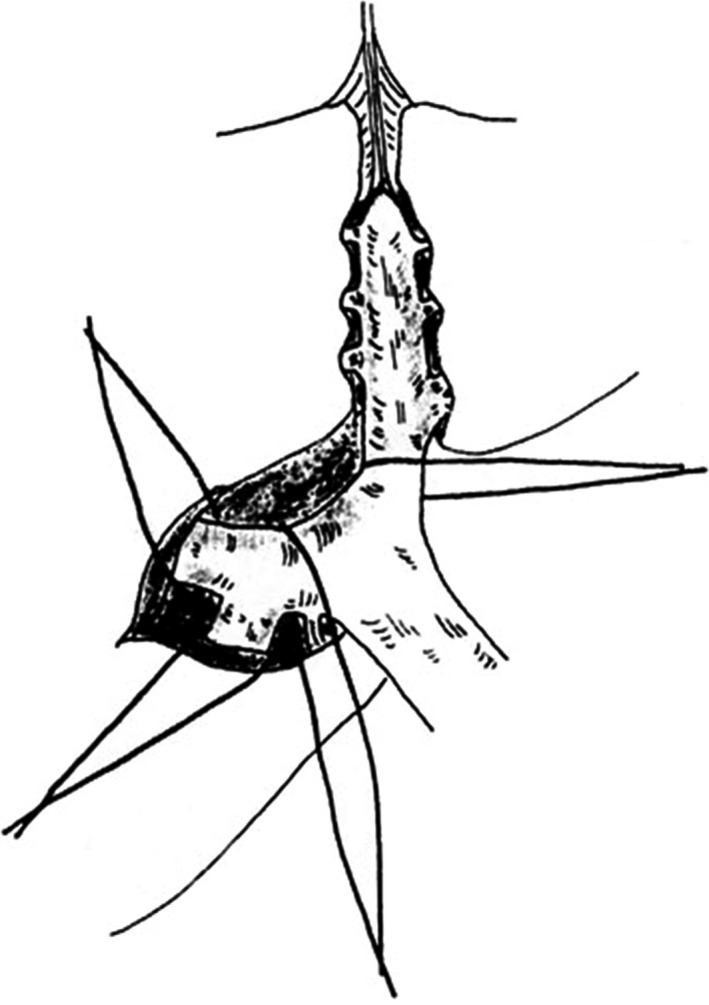
The extrafascial approach by Takasaki (Reprinted from reference^17^)

Couinaud published a report on left hepatectomy with the extrafascial approach in *Surgery* in 1985.^16^ At the same time, Takasaki published the extrafascial approach in Japanese in 1986 (Fig. 3).^17^ Takasaki reported the extrafascial approach not only for the main portal pedicle but also for the sectional portal and segmental pedicles in the left and the right liver. Therefore, various types of anatomical hepatectomy can be carried out with the extrafascial approach. Takasaki reported these procedures in English in 1990, 1998 and 2007.^2^, ^18^, ^19^

Couinaud mentions that Walaeus discovered that Glisson's capsule is derived from the vasculo‐biliary sheath and not from the peritoneum and the proper membrane of the liver.^1^ Couinaud referred to the proper membrane of the liver as Laennec's capsule. The key for completing the extrafascial approach without liver dissection is to confirm the existence of Laennec's capsule. However, Laennec's capsule is not well known because Couinaud downplayed the importance of Laennec's capsule in liver surgery in his textbook.^1^ There are very few articles regarding the membrane system of the liver. In 2008, Hayashi et al^20^ showed differences in fiber configuration between Glisson's capsule and the liver capsule. Furthermore, Sugioka recently reported that Glisson's capsule and Laennec's capsule can be divided outside and inside the liver. Therefore, the Glissonean pedicles can be separated from Laennec's capsule and not only the main portal pedicles but also the sectional and segmental pedicles can be approached at the hepatic hilus without liver dissection.^21^ This concept supports Takasaki's procedure. The magnified view in laparoscopic hepatectomy clearly shows Laennec's capsule behind the Glissonean pedicles. The concept of Laennec's capsule can facilitate the extrafascial approach in laparoscopic hepatectomy.

Several extrafascial approaches were reported in the Western world after Couinaud and Takasaki. However, the techniques were not the same as those of Couinaud and Takasaki's procedure. Galperin and Karagiulian reported that they could dissect the liver parenchyma around the main portal pedicles at the hepatic hilus and approach the hepatic pedicles with the finger fracture method.^22^ Launois and Jamieson carried out hepatotomies around the porta hepatis in two regions, posterior and anterior to the hilum, which was reported in 1992. The index finger of the surgeon was placed posteriorly through the caudate process.^23^ Batignani also reported the same procedure in 2000.^24^ These procedures led to blunt dissection of the Glissonean pedicle with forceps by Machado in sectional hepatectomy.^25^ Strasberg described the extrafascial approach as a surgeon at work in 2008. Unfortunately, however, he did not refer to Takasaki's work.^26^

The advantage of the extrafascial Glissonean approach is a simple and versatile application procedure to carry out anatomical hepatectomy. However, the disadvantages are occasional injury to the small branches from the pedicles and unachievable success if the tumor invades or is attached to the portal pedicle at the hilus. In such a case, we could change from the extrafascial approach to the intrafascial approach or the extrafascial and transfissural approach for hepatectomy.

## 5 APPROACH TO THE SECTIONAL AND SEGMENTAL GLISSONEAN PEDICLES

Bismuth reported 22 cases of segmentectomy out of almost 100 liver resections. He mentioned that segmentectomy was rarely carried out before the 1980s and described how important the primary transparenchymatous approach was for segmentectomy.^5^ Ton That Tung had the largest experience with segmentectomy before 1980. Tung reported that sectionectomy and segmentectomy with the extrafascial and transfissural approach could be carried out safely and included a large number of figures of anatomical hepatectomy in his textbook.^13^

**Figure 4:**
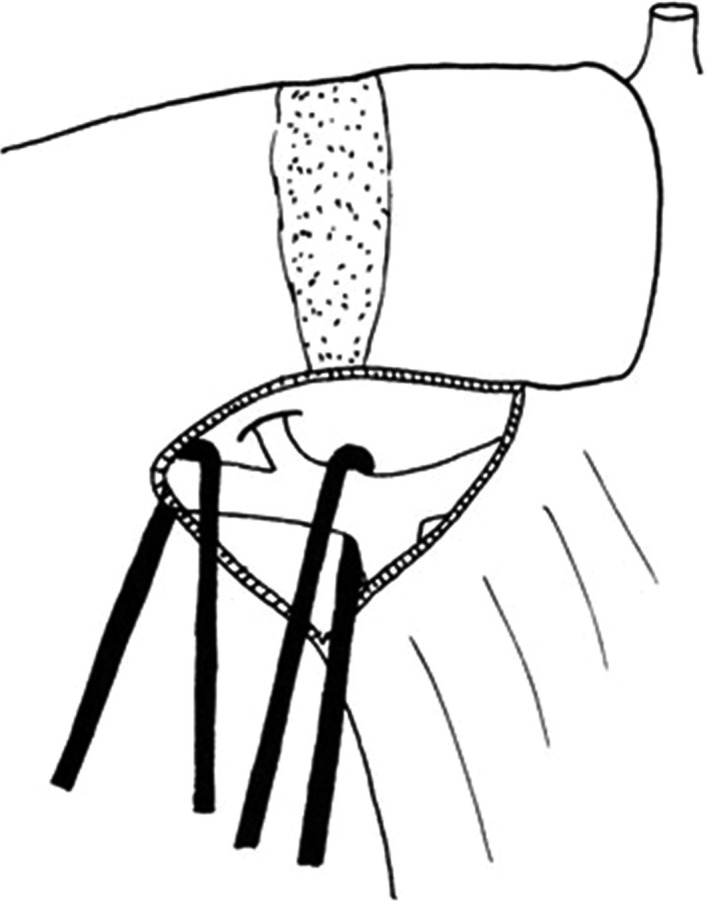
Extrafascial approach to the anterior branch of the Glissonean pedicle. The right anterior branch can be approached after strongly pulling the right main portal branch and the right posterior branch

Couinaud described not only hemihepatectomy but also sectionectomy and segmentectomy with the Glissonean pedicle approach in his textbook.^1^ The sectional pedicle could be approached after detaching the hilar plate from the liver parenchyma. Segmental pedicles to segment IV could be approached after dissecting the liver on the umbilical fissure and the segmental pedicles to segment VII could be approached after dissecting the liver on Rouviere's sulcus. Couinaud described how the right anterior pedicle and segment VIII pedicle could be approached after strongly pulling the right main portal pedicle and the right posterior pedicle (Fig. 4). This procedure was introduced by Okamoto and Hasegawa in Japan.^1^

**Figure 5:**
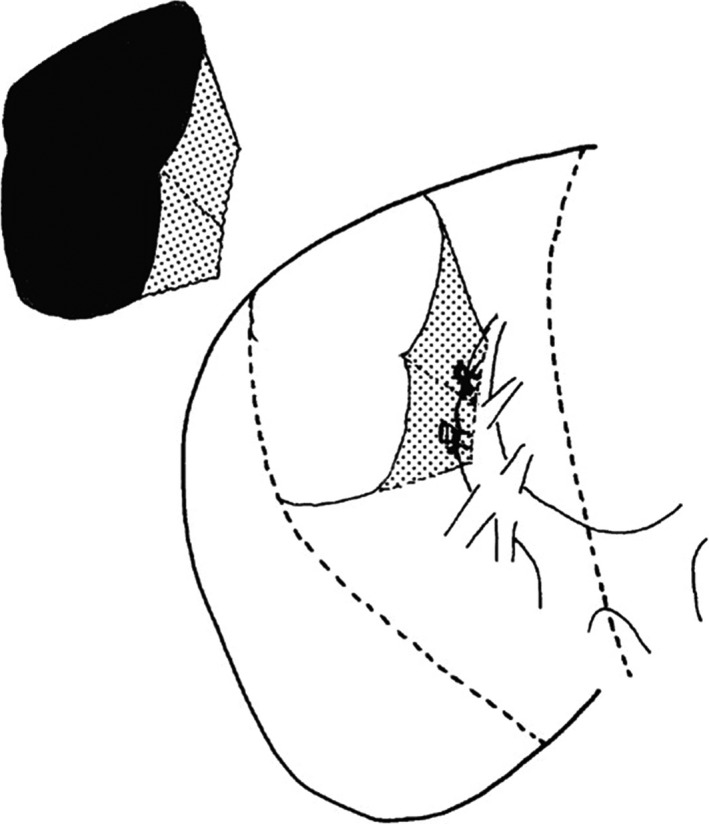
Cone unit resection. The three branches are transected selectively, by the hilar or parenchymal approach. Then the liver parenchyma may be cut on the demarcated line (Reprinted from reference^2^)

Tung, Couinaud and Takasaki recognized that several tertiary Glissonean branches enter each segment of the liver. However, there was no appropriate anatomical term for the area which was fed by one of the tertiary Glissonean branches. Takasaki proposed the new nomenclature of anatomical hepatectomy based on the Glissonean ramification at the hepatic hilus.^2^, ^17^, ^18^, ^19^ He mentioned that the area fed by a tertiary branch of the Glissonean pedicle did not correspond to one of Couinaud's segments. Therefore, Takasaki called the area fed by one of the tertiary Glissonean branches a cone unit. Therefore, cone unit resection was recognized as transection of one of the tertiary branches of the Glissonean pedicle (Fig. 5).^2^

Makuuchi et al.^27^ reported another approach for systematic subsegmentectomy in 1985. It was a procedure to puncture and inject dye to the portal pedicle for confirming the resection area under intraoperative ultrasonography. This procedure could guide the precise identification of the ligating point of the portal pedicle so that subsegmentectomy could be carried out.

## 6 SUMMARY

Various types of anatomical hepatectomy can be achieved safely by the Glissonean pedicle approach. Since the 1980s, this approach has provided new knowledge of surgical anatomy and techniques for over 30 years. Hepatobiliary surgeons should know the historical aspects and learn the concept of the Glissonean pedicle approach.

## DISCLOSURE

Conflicts of Interest: Authors declare no conflicts of interests for this article.
